# Host-specific vascular endothelial cell responses to *Angiostrongylus vasorum*: a comparative *in vitro* study in red foxes (*Vulpes vulpes*) and domestic dogs

**DOI:** 10.3389/fcimb.2025.1584663

**Published:** 2025-06-03

**Authors:** Belinda Eisenhut, Aline Wittwer, Manuela Schnyder, Andreas W. Oehm

**Affiliations:** ^1^ Institute of Parasitology, Vetsuisse Faculty, University of Zurich, Zurich, Switzerland; ^2^ Graduate School for Cellular and Biomedical Sciences, University of Bern, Bern, Switzerland

**Keywords:** canid angiostrongylosis, vascular endothelium, endothelial activation, host-specific response, host-parasite interaction, pathogenesis, comparative immunobiology

## Abstract

**Introduction:**

Canine angiostrongylosis, caused by *Angiostrongylus vasorum*, affects dogs and red foxes, with dogs developing cardiopulmonary and coagulation disorders, while foxes remain mostly subclinical.

**Methods:**

This study examined aortic endothelial cell responses from both species to *A. vasorum* adult full somatic antigen extracts, first-stage larval (L1) antigen, and adult excretory-secretory products (ESP). Differential gene expression of interleukins (IL) -6, -10, and -33, intercellular adhesion molecule 1 (ICAM-1), vascular adhesion molecule 1 (VCAM-1), endothelial selectin (E-selectin), platelet selectin (P-selectin), vascular endothelial growth factor (VEGF), and monocyte chemoattractant protein 1 (MCP-1) was assessed via reverse transcription quantitative PCR (RT qPCR) after four and 24 hours of antigen exposure.

**Results:**

Four hours post ESP stimulation, IL-10 increased in dogs (1.8-fold) but decreased in foxes (0.4-fold). IL-33 declined in both, (0.9-fold vs. 0.7-fold, respectively). VCAM-1 was upregulated more in foxes (3.5-fold vs. 1.2 in dogs). Following adult antigen exposure, P-selectin, ICAM-1, and VCAM-1 increased in fox more than in dog cells (1.4, 1.7, and 3.1-fold vs. 0.9, 0.5, and 0.7-fold, respectively). L1 antigen downregulated IL-10 and MCP-1 in dogs (0.7 and 0.8-fold) but upregulated them in foxes (2.1 and 1.1-fold). Twenty-four hours after ESP stimulation, ICAM-1 decreased in dogs (0.8-fold) but increased in foxes (1.4-fold). VCAM-1 was downregulated in dogs (0.6-fold) but upregulated in foxes (12.9-fold). Adult antigen exposure upregulated P-selectin in both species, more in foxes (4.8-fold) than in dogs (1.9-fold). ICAM-1 was downregulated in dogs (0.8-fold) but upregulated 7.5-fold in foxes. L1 antigen stimulation caused the most substantial differences between species: IL-6 was upregulated more in dogs (4.7-fold) than foxes (1.2-fold). E-Selectin was upregulated in dogs (12.8-fold) but downregulated in foxes (0.2-fold). P-selectin increased more in dogs (10.0-fold) than in foxes (1.7-fold). ICAM-1 was downregulated in dogs (0.6-fold) but upregulated in foxes (2.6-fold), as was VCAM-1 (0.7-fold and 3.1-fold). VEGF was upregulated 9.5-fold in dogs after adult antigen exposure, and 7.6-fold after L1 antigen exposure, while it remained rather unchanged in foxes (0.9-fold and 1.0-fold, respectively).

**Discussion:**

These findings corroborate that foxes have developed mechanisms for a regulated immune response following *A. vasorum* exposure, while dogs exhibit a higher pro-inflammatory reaction, contributing to severe clinical outcomes. Host-parasite co-evolution may explain differences in the pathogenesis and clinical presentation of canid angiostrongylosis.

## 1 Introduction

Canine angiostrongylosis caused by the metastrongylid nematode *Angiostrongylus vasorum* is a cardiopulmonary parasitic disease affecting domestic dogs and other canids (Guilhon, 1963; Guilhon and Cens, 1973). This parasite has a heteroxenous life cycle, with slugs and snails serving as obligate intermediate hosts, while various canid species serve as the definitive hosts (Eckert and Lämmler, 1972; Guilhon, 1963; Guilhon and Cens, 1973). *Angiostrongylus vasorum* is widely distributed across Europe (Cowie et al., 2023), in the Atlantic province of Newfoundland and Labrador in North America and in South America (Conboy, 2011). Both the incidence in dogs and the prevalence in fox populations have risen in recent decades, particularly in countries where the parasite had long been established (Gillis-Germitsch et al., 2020; Maksimov et al., 2017; Taylor et al., 2015).

Adult specimens are located in the right heart and pulmonary arteries of the definitive host where they establish chronic infections: under experimental conditions, dogs were shown to remain infected up to five years (Guilhon, 1963). During patent infections, the adult nematodes are in direct contact with the endothelium (Gillis-Germitsch et al., 2021a). First-stage larvae (L1) emerge from eggs released into the bloodstream and penetrate capillaries and alveolar epithelia inducing substantial tissue damage and inflammation (Schnyder et al., 2010).

Canine angiostrongylosis can be a fatal disease with a highly variable clinical presentation ranging from subclinical cases to evident respiratory signs, neurological disorders, and bleeding (Glaus et al., 2016; Koch and Willesen, 2009; Sigrist et al., 2017). In foxes however, clinical signs are rarely observed (Philbey, 2013; Simpson, 1996). This is particularly interesting given recent evidence of host-specific responses to *A. vasorum* infections in canids suggesting that foxes may exhibit greater tolerance to infections compared with dogs (Gillis-Germitsch et al., 2017, 2021b; Tritten et al., 2021). Based on serum proteomics, an overall more diverse immunological reaction was observed in foxes, with twice as many decreased or increased serum proteins as opposed to dogs (Gillis-Germitsch et al., 2021b; Tritten et al., 2021). These authors showed that foxes exhibit a strong innate immune response, indicating that a tightly controlled parasite tolerance may favor subclinical disease in this host species. Foxes thus appear to display fox-specific, more appropriate defense mechanisms to combat the parasite and to cope better with infection irrespective of parasite persistence. In contrast, dogs fail to mount appropriate and effective immune responses to the pathogen (Gillis-Germitsch et al., 2017, 2021b; Tritten et al., 2021).

A key aspect of this host-parasite relationship is the complex interplay between the host immune system and the plethora of helminth antigens released from the gut, the reproductive organs, the cuticula or the tegument, as well as through specific structures of the parasite (Lee et al., 2020; Maizels et al., 1982; Rhoads, 1981). These interactions happen at a vast interface throughout the host organism influencing the pathogenesis, the progression of infection, and the individual clinical presentation (Hewitson et al., 2009; Lightowlers and Rickard, 1988). In the case of *A. vasorum*, contacts between the adult parasite and the host organism particularly occur at the endothelial junction, and endothelial cells have been shown to be crucially involved in the immune response and pathogenesis of *A. vasorum* infections (Gillis-Germitsch et al., 2021a; Grob et al., 2021). The endothelium, serving as the immediate boundary between blood and tissue, is also one of the first interfaces to interact with circulating components (Opitz et al., 2007), and therefore with both excretory-secretory products of the parasite as well as with adult specimens and L1 stages. Accordingly, pathogen contact leads to the activation of endothelial cells, resulting in the upregulation of cytokines, chemokines, and adhesion molecules which trigger an abundance of pro- and anti-inflammatory responses and downstream immunological processes. This involves the production of endogenous danger signals and chemoattractants, the expression of cell adhesion molecules, and the recruitment of immune cells. However, in chronic infections, due to parasite interference, these processes may not successfully resolve infection and could even be harmful to the host (Conejeros et al., 2019; Grob et al., 2021; Maksimov et al., 2016). Chiu and Lai (2014) demonstrated that the rat lungworm *Angiostrongylus cantonensis*, a zoonotic nematode closely related to *A. vasorum*, increases blood-brain barrier permeability in infected mice through the upregulation of matrix metalloproteinase 9, suggesting an activation of endothelial cells. Similarly, it has been observed that *Dirofilaria immitis*, a heartworm infecting canids, also led to endothelial cell activation under *in vitro* conditions, thereby triggering inflammatory responses (Machado et al., 2023; Morchón et al., 2008; Mupanomunda et al., 1997). These responses promote pulmonary endarteritis and muscular hypertrophy of the arteriolar walls, which were associated with clinical manifestations such as pulmonary hypertension and right-sided heart failure (Kramer et al., 2011). Furthermore, studies in mouse models have demonstrated that infection with *Schistosoma mansoni* induces endothelial cell activation: this activation facilitated the recruitment of immune cells and the formation of granulomas, which in turn promoted chronic inflammatory responses. Ultimately, these processes resulted in pathological alterations such as liver fibrosis and portal hypertension (Figliuolo da Paz et al., 2019).

Despite the growing understanding of the role of endothelial cells in the immune response, limited knowledge exists about how these cells interact with helminth parasites, especially in a comparative context across different hosts. We hypothesized that canine and vulpine endothelial cells would exhibit distinct antigenic recognition patterns when exposed to *A. vasorum* antigens, reflecting both differences in reactions elicited by the various antigens as well as species-specific differences between the two host species. Specifically, we anticipated that fox endothelial cells would exhibit a faster and more robust activation to mitigate the harmful effects of the parasite, whereas dog endothelial cells would display a slower, less pronounced response, potentially leading to an exaggerated and detrimental activation. Furthermore, while some immunological characteristics may be shared, key differences in gene expression and immune mediator profiles were expected to contribute to species-specific variations in disease pathogenesis. Therefore, the aims of the present study were (I) to map the responses of canine and vulpine aortic endothelial cells to a panel of different antigens of *A. vasorum*, (II) to compare the time-dependent up- and downregulation of various genes related to endothelial cell activation, and (III) to comparatively identify shared and unique immunological characteristics that could translate into differential pathogenesis in both host species.

## 2 Material and methods

### 2.1 Isolation and cultivation of canine and vulpine aortic endothelial cells

For the canine model, we utilized primary endothelial cells isolated from dog aortas (Sigma-Aldrich, Buchs, Switzerland) cultured and maintained in canine aortic endothelial cell growth medium (CAEC-GM, Sigma-Aldrich, Buchs, Switzerland). Vulpine aortic endothelial cells (VAEC) were harvested from red foxes (*Vulpes vulpes*) hunted for population control in the Swiss canton of Zurich as follows: Aortas were isolated during fox necropsies within six hours of death, dissected from the hearts, and carefully cleaned from connective tissue using surgical material. Subsequently, they were transferred to sterile, ice-cold 1x phosphate-buffered saline (PBS, Gibco, Zurich, Switzerland) containing 1% (v/v) penicillin-streptomycin and amphotericin B (Anti-Anti (100x), Gibco, Zurich, Switzerland), kept on ice, and promptly processed under a sterile bench. Aortas were washed with PBS, further cleaned from connective tissue, and one end was sealed with surgical suture material. From the other end, a digestion solution prepared from 0.025 g of collagenase type II (Sigma Life Science) dissolved in 100 ml of CAEC-GM was added. Aortas were sealed on the second end and incubated at 37°C for 5–10 minutes. Subsequently, sutures were removed and the fluid from the aortic lumen was transferred to a sterile tube containing 1 ml of defined trypsin inhibitor (Gibco, Zurich, Switzerland). The aortas were then rinsed with approximately 15 ml of RPMI 1640 medium (1X) (Gibco, Zurich, Switzerland) to wash out all residual endothelial cells. The samples were centrifuged at 300 rpm for 5 minutes at room temperature, and the supernatant was carefully removed without disturbing the cell pellet. The cells were resuspended in 1 ml of CAEC-GM and subsequently seeded into T75 tissue culture flasks (TPP, Trasadingen, Switzerland) containing CAEC-GM supplemented with 1% (v/v) penicillin-streptomycin, amphotericin B, and 0.1% (v/v) gentamicin (Gibco, Zurich, Switzerland). Each flask was filled to 15 ml of medium and cultures were maintained at 37°C and 5% CO_2_ in a humidified environment. The culture medium was refreshed every three to four days. Once the cells reached confluence, the growth medium was removed followed by washing the cell layer with sterile 1x PBS. Six ml of trypsin-EDTA solution (Gibco, Zurich, Switzerland) were added, and the flask was gently swirled to ensure uniform coverage. After removing 5 ml of the trypsin-EDTA solution, the remaining volume was allowed to act on the cells for approximately one minute at 37°C. Gentle tapping of the flask helped to further detach the cells. Once most cells were detached, 5 ml of a defined trypsin inhibitor solution (Gibco, Zurich, Switzerland) was added to halt the tryptic activity. The cell suspension was transferred into a sterile tube and centrifuged at 250 x g for 5 minutes, after which the supernatant was discarded, and the cell pellet was resuspended in CryoStor C810 freezing medium (StemCell technologies, Basel, Switzerland) and kept in liquid nitrogen until further use.

A part of the cells was fixed in neutral-buffered formalin which was replaced by 70% (v/v) ethanol after 12 hours and subjected to immunohistochemical analysis. The pellet was stained with a polyclonal rabbit anti-canine antibody (dilution 1:100) specific for canine platelet endothelial cell adhesion molecule 1 (PECAM-1, CD31, Novus Biologicals, Denver, USA) following a standard horseradish peroxidase Dako autostainer (Agilent/Dako, Glostrup, Denmark) protocol with haematoxylin as counterstain. Lung tissue from a dog was used as a positive control. The primary endothelial cell cultures exhibited nearly 100% purity for CD31-positive cells (**Supplementary Figure S1**).

### 2.2 Antigen preparation

Excretory-secretory products (ESP) of *A. vasorum* were collected as previously described (Gillis-Germitsch et al., 2021a). *Angiostrongylus vasorum* adult antigen extracts were prepared from live parasites after 24 hours in culture. The specimens were washed and cut into pieces using sterile scissors. In a 2 ml tube, worms were mixed with 500 μl PBS, and four stainless steel beads (3 mm diameter, Qiagen, Hilden, Germany) were added. The tube was then placed in the Qiagen TissueLyser II (Qiagen, Hilden, Germany) at a frequency of 30 movements per second for 120 seconds. The supernatant was transferred into a cryotube, frozen in liquid nitrogen, and thawed in a 37°C water bath three times. Following this, the mixture underwent ultrasonic treatment (MSE Soniprep 150; output: 20–30, duration: 40%, 3 cycles of 25 seconds each; Medical Scientific Equipment MSE, Cholet, France) and was centrifuged at 4°C at 17,000 x g for 15 minutes. The supernatants were then transferred to a fresh tube. Throughout the process, the parasites and antigens were kept on ice unless otherwise stated.

*Angiostrongylus vasorum* L1 antigen extracts were prepared from L1 isolated from the feces of infected dogs using the Baermann-Wetzel method as previously described (Oehm and Schnyder, 2022) using kitchen towel instead of gaze to keep contamination with fecal particles at an unavoidable minimum. Larvae were repeatedly washed in sterile PBS and antigen extracts prepared as for adult specimens.

The protein concentration of all antigens was quantified using the Pierce™ BCA Protein Assay Kit (Thermo Fisher Scientific). Prior to application in cell stimulation assays, endotoxin levels were determined using the Pierce™ Chromogenic Endotoxin Quant Kit (Thermo Fisher Scientific). Only antigens with endotoxin concentrations below 0.01 ng/ml were employed in these assays. For samples with endotoxin levels exceeding this threshold, the Pierce™ High-Capacity Endotoxin Removal Resin (Thermo Fisher Scientific) was utilized to reduce the endotoxin content.

### 2.3 Cell stimulation assays

Prior to cell stimulation experiments, potential contamination of cultures with *Mycoplasma* spp. was assessed using a *Mycoplasma* gel detection kit (Biotools B&M Labs, Madrid, Spain) according to the manufacturer’s instructions. Only cultures free from *Mycoplasma* spp. were admitted to stimulation assays. All assays were conducted with cells at passage 2. Cells were thawed and seeded into a 12-well plate at a density of, with 200,000 cells/well in 2 ml of CAEC-GM. Both CAEC and VAEC were cultivated without antibiotic supplements. The cells were incubated at 37°C with 5% CO_2_ in a humidified incubator for 24 hours, allowing them to reach at least 80% confluence. A full medium exchange was performed and cultures were co-exposed to 5 µg of *A. vasorum* L1 antigen extract, 5 µg of *A. vasorum* adult antigen extract, or 5 µg of *A. vasorum* ESP, together with 1 ng of tumor necrosis factor (TNF) (Sigma-Aldrich, Buchs, Switzerland), as indicated by previous work (Gillis-Germitsch et al., 2021a). In addition, each setup included a positive control using 1µl of a 1:100 dilution of phorbol 12-myristate 12-acetate (PMA) and 10µl of a 1:100 dilution of ionomycin (Iono) (both from Sigma-Aldrich, Buchs, Switzerland), as well as a negative control with 1 ng of TNF only. Cells were collected at four and 24 hours post-stimulation for further analysis.

### 2.4 Gene expression analysis by quantitative reverse transcription PCR analysis

After removing medium supernatants, full mRNA was isolated from cells using the RNeasy^®^ Mini Kit (Qiagen, Hilden, Germany) following the manufacturer’s protocol. The cell monolayer was thoroughly scraped off using a 24 cm cell scraper (TPP, Trasadingen, Switzerland). RNA concentration and purity were assessed using the Nanodrop OneC (Thermo Fisher Scientific, Zurich, Switzerland). The RNA was stored at -80°C until further processing.

Genomic DNA was eliminated in accordance with the instructions from the DNA-free DNA Removal Kit (Thermo Fisher Scientific, Zurich, Switzerland). Complementary DNA (cDNA) was synthesized using the Maxima H minus cDNA Synthesis Master Mix (5x) (Thermo Scientific, Zurich, Switzerland) and reverse transcription was performed on a C1000 Thermal Cycler (Bio-Rad Laboratories, Munich, Germany) according to the manufacturer’s protocol at 25°C for 10 minutes, at 50°C for 15 minutes and at 85°C for 5 minutes. Subsequently, the cDNA was quantified using the Nanodrop OneC (Thermo Fisher Scientific, Zurich, Switzerland).

For the qPCR analysis, the PowerUp SYBR Green Master Mix (Applied Biosystems, Zurich, Switzerland) was used. A reaction mixture was prepared by combining the SYBR Green Master Mix, 1 µM of each forward and reverse primer (**Table 1**), and nuclease-free water. To assess endothelial activation, we selected a panel of key markers involved in inflammation, adhesion, and vascular remodeling. Interleukins (IL) -6, -10, and -33 were chosen to evaluate pro- and anti-inflammatory cytokine responses, while intercellular adhesion molecule 1(ICAM-1), vascular cell adhesion molecule 1 (VCAM-1), endothelial selectin (E-selectin), and platelet selectin (P-selectin) were included as critical adhesion molecules facilitating leukocyte recruitment. Vascular endothelial growth factor (VEGF) was analyzed for its role in endothelial permeability and angiogenesis, and monocyte chemoattractant protein 1 (MCP-1) was selected due to its importance in monocyte chemotaxis (Krishnaswamy et al., 1999; Moussion et al., 2008; Zegeye et al., 2018). Canine glyceraldehyde-3-phosphate dehydrogenase (GAPDH) was used as a housekeeping gene and internal control. For the qPCR analysis, technical triplicates were included for each sample. For dog endothelial cells, three independent experiments were conducted, representing three biological replicates. For the fox material, we used cells from three different donors, with each donor’s stimulation experiment set up in duplicate. cDNA samples, at a final concentration of no less than 5 ng/µl, were dispensed into a MicroAmp Optical 96-well reaction plate (Applied Biosystems, Zurich, Switzerland). Subsequently, the appropriate reaction mixture was added to each well to achieve a final volume of 10 µl. Amplification was performed using a QuantStudio 7 Flex (Applied Biosystems, Zurich, Switzerland), following the manufacturer’s standard thermal cycling conditions, which included an initial step of 2 minutes at 50°C and 2 minutes at 95°C, followed by 40 cycles of 15 seconds at 95°C, 15 seconds at the primer-specific annealing temperature (**Table 1**), and 1 minute at 72°C.

**Table 1:** Primer sequences, references, and annealing temperatures for the target genes analyzed.

Genes	Primer sequences	Annealing temperature	Reference
IL-6	Forward: 5’-CCCACCAGGAACGAAAGAGA-3’ Reverse: 5’CTTGTGGAGAGGGAGTTCATAGC-3’	55.7°C	(Yu et al., 2010)
IL-10	Forward: 5’-CCCGGGCTGAGAACCACGAC-3’ Reverse: 5’-AAATGCGCTCTTCACCTGCTCCAC-3’	63°C	(Veenhof et al., 2010)
IL-33	Forward: 5’-GTACTTTATGCAACTGCGTTCTGG-3’ Reverse: 5’-CAGACATTGCTTTCTGCACTTTTC-3’	60°C	(Asahina et al., 2018)
ICAM-1	Forward: 5’-CAGGGTTGCCAGGTACAGTT-3’ Reverse: 5’-AGTATGGGCTCAGTGGGTTG-3’	53.4°C	(Asahina et al., 2018)
VCAM-1	Forward: 5’-TCCATCGTGGAGGAAGGTAG-3’ Reverse: 5’-CAGCCTGGTTAATCCCTTCA-3’	53.4°C	(Grob et al., 2021)
E-selectin	Forward: 5’-TGGCTTCAGAGGTCTCAGGT-3’ Reverse: 5’-TCAAAGCACTGCACTCAACC-3’	55.7°C	(Grob et al., 2021)
VEGF	Forward: 5’-GTACCTCCACCATGCCAAGT-3’ Reverse: 5’-AATAGCTGCGCTGGTAGACG-3’	55.7°C	(van Albada et al., 2008)
MCP-1	Forward: 5’-GAGTCACCAGCAGCAAGTGT-3’ Reverse: 5’-TGGGTTTGGCTTTTCTTGTC-3’	55.7°C	(Teoh et al., 2023)
P-selectin	Forward: 5’-CTGCACCAATCTGCAAAGC-3’ Reverse: 5’-ATGAGGGCTGGACACTGAAC-3’	55.7°C	(Wood et al., 2009)
GAPDH	Forward: 5’-GGAGAAAGCTGCCAAATATG-3’ Reverse: 5’-ACCAGGAAATGAGCTTGACA-3’	55.5°C	(Park et al., 2013)

IL-6, Interleukin 6; IL-10, Interleukin 10; IL-33, Interleukin 33; ICAM-1, Intercellular adhesion molecule 1; VCAM-1, Vascular cell adhesion molecule 1; E-selectin, Endothelial-selectin; VEGF, Vascular endothelial growth factor; MCP-1, Monocyte chemoattractant protein 1; P-selectin, Platelet-selectin; GAPDH, Glyceraldehyde 3-phosphate dehydrogenase.

### 2.5 Data analysis

Relative expression analysis was manually performed using Pfaffl’s Augmented ΔΔCt method (Pfaffl, 2001), where the comparative cycle threshold (Ct) values of the samples were compared to the negative control and normalized to the housekeeping gene. To present data in a more interpretable form, relative fold changes were produced using the 2^-ΔΔCT^ calculation (Livak and Schmittgen, 2001). Primer efficiency was evaluated by qPCR using a five-step, tenfold serial dilution of cDNA. The same reagents and conditions were applied as in the preceding analyses. A standard curve of Ct values versus the logarithm of initial template concentrations was used to calculate primer efficiencies (E) (Meuer et al., 2012). Efficiencies ranged from 90% to 110%, which is consistent with the specifications provided by the European Network of GMO Laboratories (ENGL, 2015), and calculations were performed using Microsoft Excel Version 16.89.1. Throughout the analyses, statistical significance was set at p ≤ 0.05. Normality of the data was assessed via density plots and the Shapiro-Wilk test. Comparisons between antigens were conducted at the species and time-point-of-stimulation level, i.e. for the dog model at four hours and at 24 hours as well as for the fox model at four hours and at 24 hours. Normally distributed data were explored by one-way analysis of variance (ANOVA) with Tukey’s honestly significant difference (HSD) test for *post-hoc* analysis to address the issue of multiple comparisons in cases where ANOVA yielded statistically significant results. Non-normal data were analyzed via Kruskal-Wallis test followed by a pairwise Wilcoxon signed-rank test to identify differences. P-values were adjusted using the Bonferroni multiple testing correction method.

Comparisons between four and 24 hours of stimulation were performed at the species level within antigen, e.g., ESP 4h vs. ESP 24h for the dog model. Due to the dependence of samples, matched samples were compared by a paired t-test (for normally distributed data) or by a Wilcoxon signed-rank test (for non-normally distributed data).

Comparisons between the dog and the fox model were carried out at the time-point-of-stimulation level, i.e. at four hours and at 24 hours, and within antigens, applying Student’s t-test (normally distributed data) or Mann-Whitney U-test (non-normally distributed data), respectively. Calculations were performed in Microsoft Excel Version, R software for statistical computing version 4.3.2 (R Core Team, 2023), and GraphPad Prism version 9.4.1.

## 3 Results

### 3.1 Gene expression changes after stimulation with *A. vasorum* antigens

The expression levels of IL-6, IL-10, IL-33, VEGF, MCP-1, E-selectin, P-selectin, ICAM-1, and VCAM-1 were analyzed in endothelial cells from dogs and foxes following stimulation with ESP, adult antigen, and L1 antigen of *A. vasorum*. Gene expression was measured at four and 24 hours after stimulation to capture time-dependent changes in regulation. **Table 2** summarizes the mean fold changes in gene expression for canine endothelial cells, while **Table 3** provides the corresponding data for fox cells.

**Table 2:** Mean fold changes in the expression of the target genes four and 24 hours after stimulation with *Angiostrongylus vasorum* excretory-secretory products, adult antigen, and L1 antigen in dogs.

	4 hours			24 hours		
Target gene	ESP	Adult antigen	L1 antigen	ESP	Adult antigen	L1 antigen
IL-6	1.05	0.79	1.18	1.08	1.45	4.68
IL-10	1.76	0.66	0.71	0.85	0.41	0.49
IL-33	0.91	0.47	0.88	0.80	0.19	0.74
VEGF	0.63	0.65	0.67	1.00	9.48	7.62
MCP-1	0.97	0.93	0.78	0.70	1.26	0.77
E-selectin	0.98	0.75	1.11	1.00	1.33	12.79
P-selectin	1.33	0.86	0.98	0.81	1.87	9.99
ICAM-1	0.92	0.53	0.94	0.75	0.83	0.57
VCAM-1	1.21	0.70	1.18	0.59	1.25	0.72

Mean fold changes are presented relative to the housekeeping gene glyceraldehyde 3-phosphate dehydrogenase (GAPDH). Values greater than 1 indicate upregulation of gene expression, while values less than 1 indicate downregulation. IL-6, Interleukin 6; IL-10, Interleukin 10; IL-33, Interleukin 33; ICAM-1, Intercellular adhesion molecule 1; VCAM-1, Vascular cell adhesion molecule 1; E-selectin, Endothelial-selectin; VEGF, Vascular endothelial growth factor; MCP-1, Monocyte chemoattractant protein 1; P-selectin, Platelet-selectin; ESP, Excretory-secretory products; L1, *Angiostrongylus vasorum* first-stage larvae.

**Table 3:** Mean fold changes in the expression of the target genes four and 24 hours after stimulation with *Angiostrongylus vasorum* excretory-secretory products, adult antigen, and L1 antigen in foxes.

	4 hours			24 hours		
Target gene	ESP	Adult antigen	L1 antigen	ESP	Adult antigen	L1 antigen
IL-6	0.77	0.92	1.30	1.08	1.14	1.24
IL-10	0.40	0.56	2.10	0.44	0.54	1.08
IL-33	0.65	0.81	1.39	0.87	1.59	1.52
VEGF	0.74	0.99	1.12	0.62	0.91	1.04
MCP-1	1.02	1.17	1.07	0.94	1.21	1.00
E-selectin	0.69	1.18	3.90	1.06	1.13	0.23
P-selectin	0.93	1.36	1.43	1.63	4.78	1.66
ICAM-1	0.88	1.65	0.53	1.44	7.50	2.61
VCAM-1	3.46	3.06	2.74	12.88	2.07	3.10

Mean fold changes are presented relative to the housekeeping gene glyceraldehyde 3-phosphate dehydrogenase (GAPDH). Values greater than 1 indicate upregulation of gene expression, while values less than 1 indicate downregulation. IL-6, Interleukin 6; IL-10, Interleukin 10; IL-33, Interleukin 33; ICAM-1, Intercellular adhesion molecule 1; VCAM-1, Vascular cell adhesion molecule 1; E-selectin, Endothelial-selectin; VEGF, Vascular endothelial growth factor; MCP-1, Monocyte chemoattractant protein 1; P-selectin, Platelet-selectin; ESP, Excretory-secretory products; L1, *Angiostrongylus vasorum* first-stage larvae.

### 3.2 Comparison of antigens

#### 3.2.1 Comparison of antigens in dogs after four hours of stimulation

Four hours after antigen stimulation, differences in IL-10 expression were observed in dogs across the three antigens. Compared with L1 antigen, which appeared to result in a downregulation of IL-10 after four hours (mean fold change 0.71), IL-10 expression was upregulated after stimulation with ESP, with a mean fold change of 1.76 (p = 0.003). Interleukin-10 expression was also downregulated following stimulation with adult antigen (mean fold change of 0.66) which was more pronounced than after L1 stimulation (mean fold change 0.71, p = 0.01).

#### 3.2.2 Comparison of antigens in dogs after 24 hours of stimulation

Twenty-four hours post-stimulation, differences between antigens were observed regarding the expression of ICAM-1 and VCAM-1. Intercellular adhesion molecule 1 expression was downregulated for all antigens with a less pronounced decrease (p = 0.05) in expression after adult antigen exposure (mean fold change 0.83) compared with L1 antigen (mean fold change 0.57). Mean fold change after ESP exposure was 0.75. Vascular cell adhesion molecule 1 expression also exhibited differences. VCAM-1 was upregulated with a mean fold change of 1.25 following adult antigen stimulation compared with ESP stimulation (mean fold change: 0.59, p = 0.001) and L1 antigen (mean fold change of 0.72, p = 0.004).

#### 3.2.3 Comparison of antigens in foxes after four hours of stimulation

In foxes, IL-6 and E-selectin were differentially expressed depending on antigen type 4 hours after antigen exposure. IL-6 was upregulated after stimulation with L1 antigen (mean fold change: 1.30) compared with a downregulation following ESP (mean fold change of 0.77, p = 0.003) and adult antigen (mean fold change of 0.92, p = 0.01). E-selectin was considerably upregulated after L1 antigen stimulation (mean fold change 3.90). Adult antigen elicited a slight upregulation of E-selectin (mean fold change 1.18, p = 0.01), whereas ESP induced a downregulation (mean fold change 0.69, p = 0.001).

#### 3.2.4 Comparison of antigens in foxes after 24 hours of stimulation

Twenty-four hours after antigen stimulation in foxes, differences were observed in ICAM-1 and VCAM-1 expression depending on antigen. ICAM-1 was upregulated after stimulation with ESP (mean fold change 1.44) and even more pronounced after exposure to adult antigen (mean fold change 7.50, p = 0.02). L1 antigen induced an upregulation of 2.61 folds. VCAM-1 was strongly upregulated following ESP stimulation (mean fold change 12.88) compared with exposure to adult antigen (mean fold change 2.07, p = 0.001) and to L1 antigen (mean fold change 3.10, p = 0.004).

### 3.3 Comparison of time points

#### 3.3.1 Gene expression at four and 24 hours in dogs

**Figure 1:**
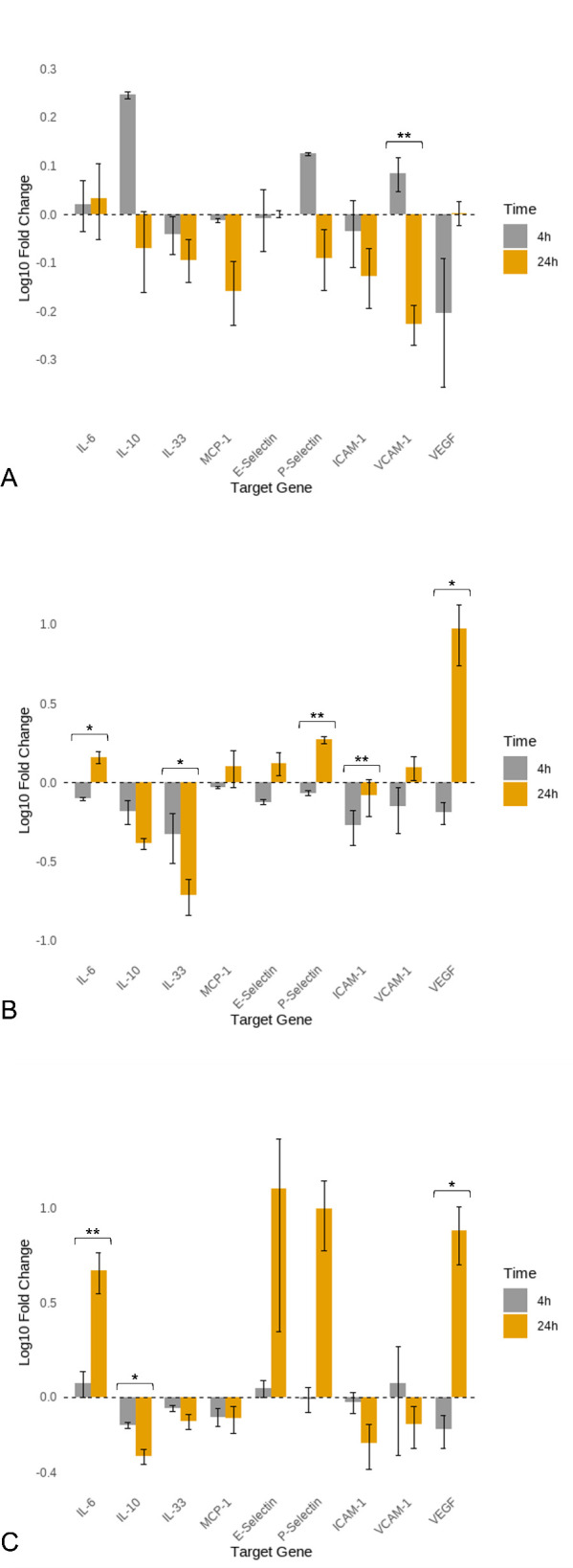
Log10 mean fold changes in gene expression of canine aortic endothelial cells at 4 and 24 hours of stimulation with *Angiostrongylus vasorum* antigens. **(A)** Excretory-secretory products (ESP); **(B)** Adult antigen; **(C)** L1 antigen. L1: *Angiostrongylus vasoru*m first-stage larvae; AG: antigen. Statistical significance is indicated by asterisks: p ≤ 0.05 (*), p ≤ 0.01 (**).

**Figure 1A** shows the mean fold changes in gene expression for the analysed genes at four- and 24-hours after ESP stimulation. A tendential transient upregulation of IL-10 was observed at four hours (mean fold change 1.76), which was instead downregulated at 24 hours (mean fold change 0.85, p = 0.08). A similar pattern was observed for P-selectin, which was upregulated at four hours (mean fold change 1.33) but markedly downregulated by 24 hours (mean fold change 0.81, p = 0.08). VCAM-1 was upregulated at four hours (mean fold change 1.21) followed by nearly a twofold downregulation at 24 hours (mean fold change 0.59, p = 0.01).

**Figure 1B** presents the mean fold changes in gene expression for the analysed genes at four and 24 hours after stimulation with adult antigen. IL-6 was downregulated at four hours (mean fold change 0.79) but upregulated at 24 hours (mean fold change 1.45, p = 0.02). IL-33 was downregulated at four hours (mean fold change 0.47) and showed further downregulation at 24 hours (mean fold change 0.19, p = 0.05). P-selectin was downregulated at four hours (mean fold change 0.86) and upregulated at 24 hours (mean fold change 1.87, p < 0.001). ICAM-1 was downregulated at both time points, with the downregulation being more pronounced at four hours (mean fold change 0.53) than at 24 hours (mean fold change 0.83, p = 0.002). VEGF was strongly downregulated at four hours (mean fold change 0.65), followed by extreme upregulation at 24 hours (mean fold change 9.48, p = 0.03).

**Figure 1C** illustrates the mean fold changes in gene expression for the analysed genes at four and 24 hours after L1 antigen stimulation. IL-6 was upregulated at four hours (mean fold change 1.18), with a further increase observed at 24 hours (mean fold change 4.68, p = 0.01). IL-10 was downregulated at both four (mean fold change 0.71) and 24 hours (mean fold change 0.49), with the downregulation being more pronounced at 24 hours (p = 0.02). VEGF expression was downregulated at four hours (mean fold change 0.67) but showed marked upregulation at 24 hours (mean fold change 7.62, p = 0.02). E-selectin showed slight upregulation at four hours (mean fold change 1.11), which increased substantially by 24 hours (mean fold change 12.79); however, this difference was not statistically significant (p = 0.08).

#### 3.3.2 Gene expression at four and 24 hours in foxes

**Figure 2:**
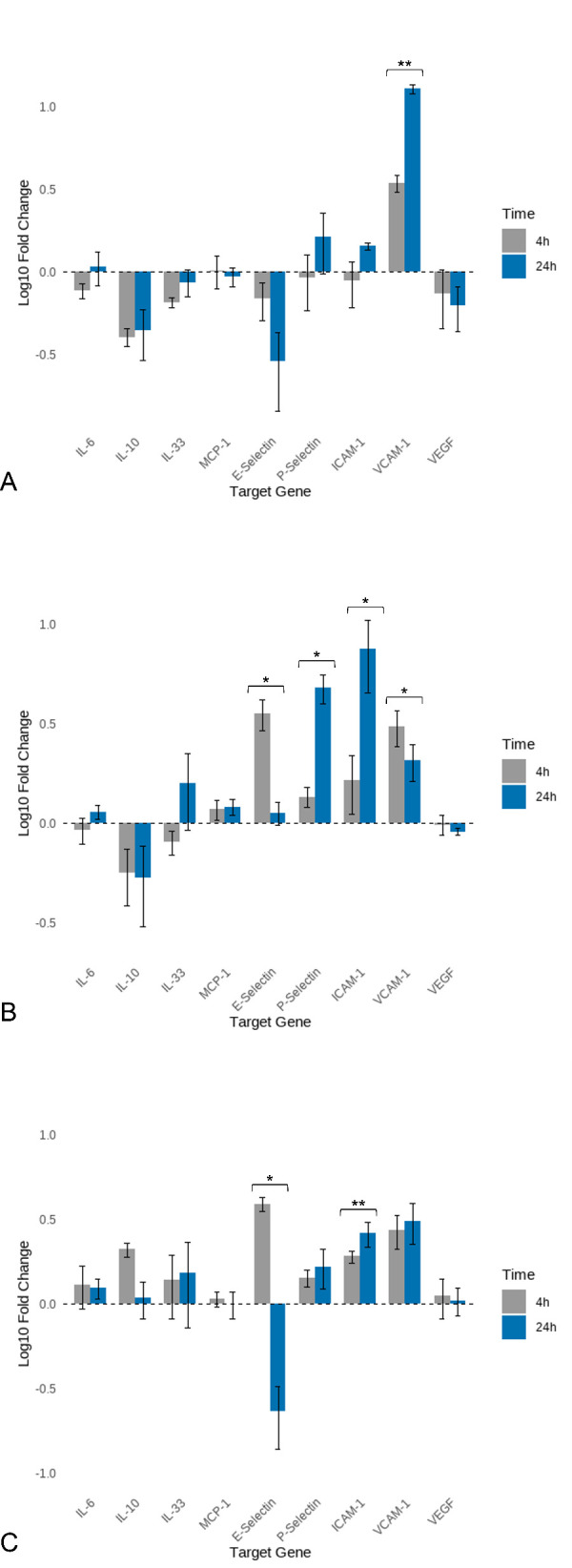
Log10 mean fold changes in gene expression of vulpine aortic endothelial cells at four and 24 hours of stimulation with *Angiostrongylus vasorum* antigens. **(A)** Excretory-secretory products (ESP); **(B)** Adult antigen; **(C)** L1 antigen. L1: *Angiostrongylus vasorum* first-stage larvae; AG: antigen. Statistical significance is indicated by asterisks: p ≤ 0.05 (*), p ≤ 0.01 (**).

**Figure 2A** shows the mean fold changes (log10) in gene expression for the analysed genes at four and 24 hours following ESP stimulation in foxes. VCAM-was upregulated at four hours (mean fold change 3.46), and this upregulation increased further at 24 hours (mean fold change 12.88, p = 0.01).

**Figure 2B** presents the mean fold changes in gene expression for the analysed genes at four and 24 hours following stimulation with adult antigen in foxes. P-selectin was upregulated at both time points, with higher upregulation observed at 24 hours (mean fold change 4.78) than at four hours (mean fold change 1.36, p = 0.03). ICAM-1 was upregulated at four hours (mean fold change 1.65), with an increase in upregulation at 24 hours (mean fold change 7.50, p = 0.04). VCAM-1 was upregulated at four hours (mean fold change 3.06) and remained upregulated at 24 hours (mean fold change 2.07), although to a lesser extent (p = 0.04). E-selectin showed no notable change at four hours (mean fold change 1.18), with a similar level at 24 hours (mean fold change 1.13); however, this difference was statistically significant (p = 0.05).

**Figure 2C** illustrates the mean fold changes in gene expression for the analysed genes at four and 24 hours after stimulation with L1 antigen in foxes. E-selectin was upregulated at four hours (mean fold change 3.90) and downregulated by a factor of four at 24 hours (mean fold change 0.23, p = 0.02). Conversely, ICAM-1 was downregulated at four hours (mean fold change 0.53) and upregulated at 24 hours (mean fold change 2.61, p = 0.001).

### 3.4 Comparison between species

#### 3.4.1 Differences in gene expression between dogs and foxes four hours after antigen stimulation

**Figure 3:**
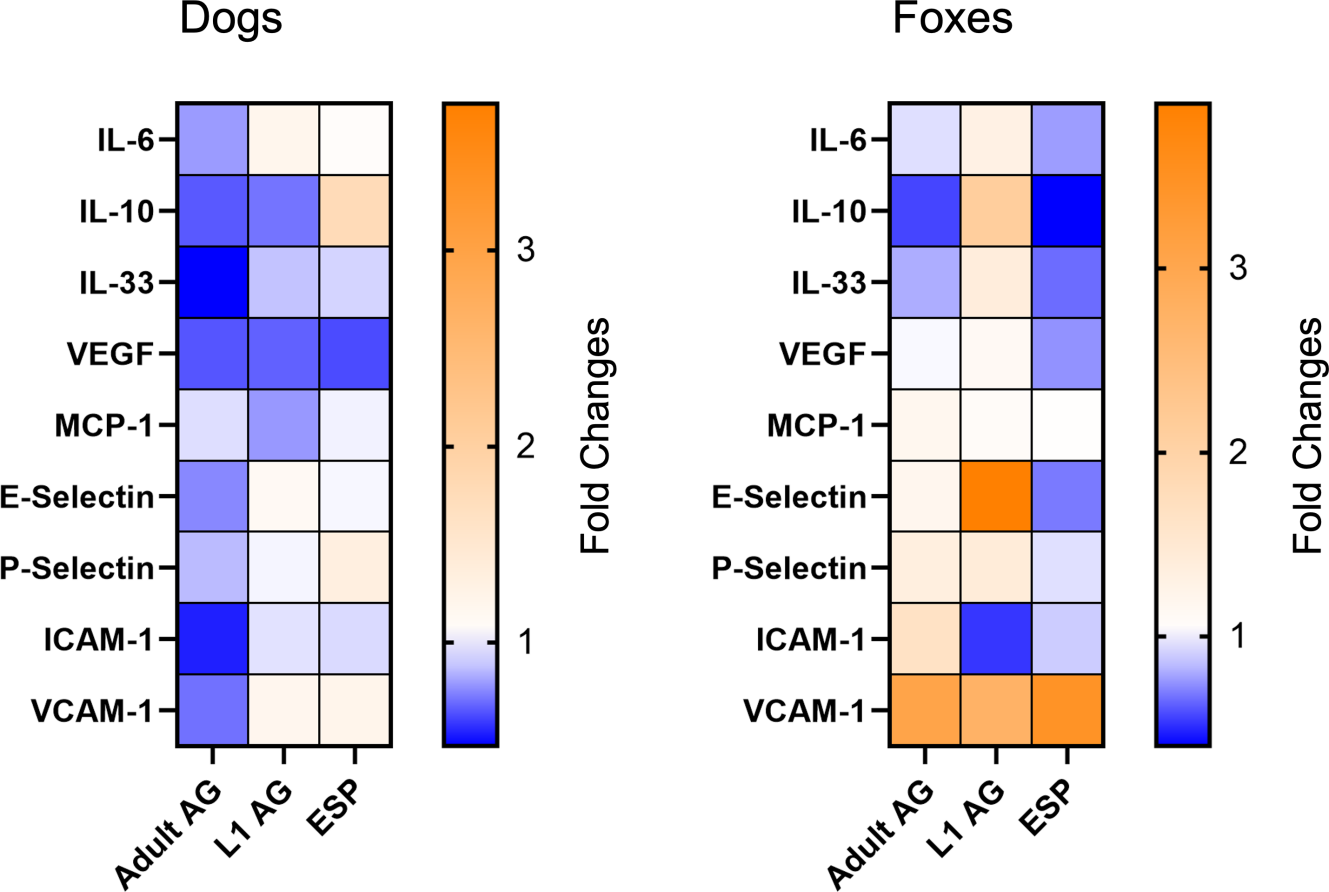
Relative gene expression changes after four hours of stimulation in dog and fox aortic endothelial cells. The data are presented as mean fold changes. Fold change values greater than 1 represent upregulation and values below 1 indicate downregulation. AG, antigen; ESP, excretory-secretory products; L1, *Angiostrongylus vasorum* first-stage larvae.

**Figure 3** displays the mean fold changes in gene expression for the three analyzed antigens in dogs and foxes four hours post-antigen stimulation, presented as heatmaps. An initial examination indicates that a more diverse relative gene expression following antigen exposure was present in the fox cells compared with the dog cells, with target genes mainly being upregulated, whereas in dogs downregulation of gene expression was more frequently observed.

Following stimulation with the ESP antigen, IL-10 was upregulated in dogs (mean fold change 1.76), while it was downregulated in foxes (mean fold change 0.40, p = 0.01). IL-33 was downregulated in both species, with a stronger downregulation observed in foxes (mean fold change 0.65) compared with dogs (mean fold change 0.91, p = 0.05). VCAM-1 expression was increased in both species, although this effect was more pronounced in foxes (mean fold change 3.46) than in dogs (mean fold change 1.21, p = 0.003).

After stimulation with adult antigen, P-selectin was downregulated in dogs (mean fold change 0.86) but upregulated in foxes (mean fold change 1.36, p = 0.05). ICAM-1 expression decreased twofold in dogs (mean fold change 0.53), while it increased in foxes (mean fold change 1.65, p = 0.05). VCAM-1 followed a comparable pattern, being downregulated in dogs (mean fold change 0.70) while strongly upregulated in foxes (mean fold change 3.06, p = 0.04).

After exposure to L1 antigen, IL-10 was downregulated in dogs (mean fold change 0.71), while it was upregulated in foxes (mean fold change 2.10, p = 0.003). MCP-1 exhibited downregulation in dogs (mean fold change 0.78), while in foxes it remained close to the baseline level (mean fold change 1.07, p = 0.05). E-selectin was upregulated in both species, but this upregulation was stronger in foxes (mean fold change 3.90) than in dogs (mean fold change 1.11. p < 0.001). While ICAM-1 expression remained almost unchanged in dogs (mean fold change 0.94), it was downregulated twofold in foxes (mean fold change 0.53, p = 0.01). Finally, VCAM-1 was upregulated in both species, though the upregulation was more pronounced in foxes (mean fold change 2.74) than in dogs (mean fold change 1.18, p = 0.09).

#### 3.4.2 Differences in gene expression between dogs and foxes 24 hours after antigen stimulation

**Figure 4:**
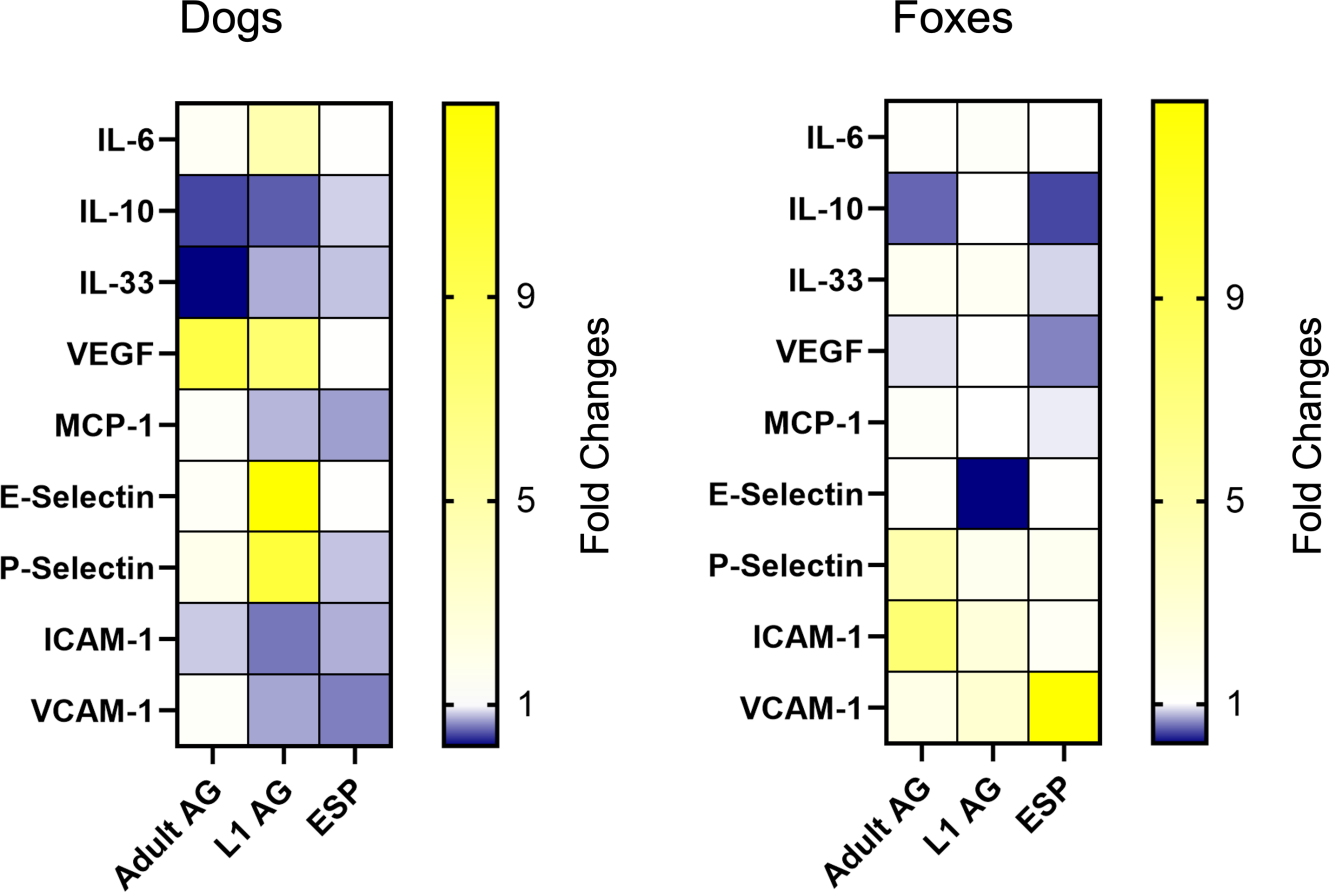
Relative gene expression changes after 24 hours of stimulation in dog and fox aortic endothelial cells. The data are presented as mean fold changes. Fold change values greater than 1 represent upregulation and values below 1 indicate downregulation. AG, antigen; ESP, excretory-secretory products; L1, *Angiostrongylus vasorum* first-stage larvae.

**Figure 4** presents the mean fold changes in gene expression for the three analyzed antigens in dogs and foxes 24 hours after antigen stimulation, displayed as heatmaps. An initial examination reveals that a greater number of genes in dogs exhibit changes in expression, either through upregulation or downregulation, compared with foxes. While some genes in foxes are also up- or downregulated, this occurs in fewer genes, with many remaining at their baseline level, indicating that they are neither upregulated nor downregulated.

Twenty-four hours after ESP stimulation, differences were observed in the gene expression of ICAM-1 and VCAM-1 between the species. In dogs, ICAM-1 was downregulated (mean fold change 0.75), while it was upregulated in foxes (mean fold change 1.44, p = 0.04). VCAM-1 was nearly twofold downregulated in dogs (mean fold change 0.59), while in foxes, it exhibited more than a 12-fold upregulation (mean fold change 12.88, p < 0.001).

Stimulation with the adult antigen revealed differences in the expression of VEGF, P-selectin, and ICAM-1. In dogs, VEGF was upregulated nearly tenfold 24 hours after stimulation (mean fold change 9.48), while in foxes the mean fold change remained close to one (mean fold change 0.91), indicating minimal change (p = 0.04). P-Selectin was upregulated in both species, though this upregulation was more pronounced in foxes (mean fold change 4.78) than in dogs (mean fold change 1.87, p = 0.03). ICAM-1 was slightly downregulated in dogs (mean fold change 0.83), while in foxes, it exhibited a more than sevenfold upregulation (mean fold change 7.50, p = 0.03).

Stimulation with the L1 antigen elicited the most pronounced differences in gene expression between both species. IL-6 was upregulated in both species, but the upregulation was stronger in dogs (mean fold change 4.68) than in foxes (mean fold change 1.24, p = 0.02). VEGF was upregulated more than sevenfold in dogs (mean fold change 7.62), while it remained unchanged in foxes (mean fold change 1.04, p = 0.02). E-selectin showed an almost 13-fold upregulation in dogs (mean fold change 12.79), whereas foxes exhibited a fivefold downregulation (mean fold change 0.23, p = 0.05). P-selectin was upregulated in both species, although the upregulation in foxes (mean fold change 1.66) was minimal compared to the tenfold upregulation observed in dogs (mean fold change 9.99, p = 0.04). ICAM-1 was downregulated twofold in dogs (mean fold change 0.57) but showed a pronounced upregulation in foxes (mean fold change 2.61, p = 0.01). A similar pattern was observed for VCAM-1, which was downregulated in dogs (mean fold change 0.72) but upregulated in foxes (mean fold change 3.10, p = 0.02).

## 4 Discussion

Parasitism is a widespread and incredibly successful mode of life in as much as half of all known species on this planet (Brooks and Hoberg, 2006; Hoberg et al., 2008; Poulin, 2014). Parasites have evolved into sophisticated organisms that are able to successfully infect virtually every other life form. Even though parasites are assumed to exert a certain degree of damage to their respective host, they are not always malignant. Commonly, parasites have optimally adapted to their phylogenetically original hosts. Distinct host-parasite associations with phylogenetic congruence between parasite and host lend support to idea of co-evolution between host and parasite lineages (Braga et al., 2020; McKee et al., 2019). Although infecting different species, differential host preference, pathogenesis, and parasitic virulence can be attributed to intrinsic host characteristics and varying parasite-host co-emergence (Medeiros et al., 2013). Accordingly, host immune response varies among host species and some hosts may adapt and be able to tolerate the pathogen mitigating destructive potential of the parasite (Gupta et al., 2020). In their prime hosts, parasites evolve towards minimal virulence, and tolerant host species represent optimal reservoirs for the parasite to thrive and circulate in the population. In situations where host switch occurs from a reservoir host to more vulnerable, less tolerant, and phylogenetically different hosts, the pathogen may be maladapted to the new host and produce fulminating and often fatal disease (Brooks and Hoberg, 2006; Dobson and Foufopoulos, 2001; Knutie et al., 2016).

*Angiostrongylus vasorum* represents an excellent example of a parasite encompassing a variability in pathogenetic potential and virulence in different host species (Jefferies et al., 2009; Cowie et al., 2023; Morgan et al., 2012). Evidence suggests that *A. vasorum* originally spread worldwide alongside its canid hosts and subsequently evolved into genetically distinct populations adapted to different host species (Gerrikagoitia et al., 2010; Jefferies et al., 2009; Morgan et al., 2012). In Europe, red foxes represent the principal sylvatic reservoir host for *A. vasorum*, phylogenetically associated with the parasite and able to carry high worm burdens up to 88 worm specimens per fox (Gillis-Germitsch et al., 2020; Jefferies et al., 2010; Tayyrov et al., 2021). Clinical signs of infection are largely absent in foxes (Simpson, 1996; Woolsey et al., 2017), whereas angiostrongylosis can be a devastating, potentially fatal condition in dogs (Glaus et al., 2016; Sigrist et al., 2017; Staebler et al., 2005). Even though both host species display common reactions to the infection, including similar pathohistological alterations in infected lung tissue (Poli et al., 1991; Schnyder et al., 2010), key differences exist reflecting host-specific patterns and intrinsic adaptation to the parasite with foxes appearing to display fox-specific, more appropriate and effective defense mechanisms to combat the parasite and to cope better with infection irrespective of parasite persistence (Gillis-Germitsch et al., 2021a, 2021b; Tritten et al., 2021). For example, while circulating parasite antigen can be detected in a comparable manner across species, host-specific differences exist in antibody responses. Foxes exhibit highly variable individual antibody courses, with antibody levels declining later in infection, whereas dogs maintain persistently high levels of non-protective serum antibodies (Gillis-Germitsch et al., 2021b; Schucan et al., 2012).

In our investigations, distinct differences in the gene expression of canine and vulpine endothelial cells were observed, depending on the type of antigen, the stimulation time point, and most remarkably the host species. At four hours, fox cells displayed a more varied gene expression response, predominantly through upregulation, while dogs exhibited more frequent gene downregulation. By 24 hours, gene expression changes were more widespread in dogs, with more genes being up- or downregulated, whereas in foxes, fewer genes were affected, with many remaining at baseline.

Specifically, four hours after stimulation with ESP, the anti-inflammatory cytokine IL-10 (Tedgui and Mallat, 2001) was upregulated in canine endothelial cells but downregulated in fox endothelial cells. IL-33 was downregulated in both species, with a more pronounced decrease in fox endothelial cells. In contrast, VCAM-1 was upregulated in both species, with a stronger response in fox endothelial cells. These results suggest that already four hours after ESP stimulation, fox endothelial cells exhibit a stronger, yet moderate pro-inflammatory response than canine endothelial cells. Overall, both dogs and foxes showed only minor changes in gene expression after ESP stimulation, indicating that ESP likely induces a mild response while avoiding a strong inflammatory reaction. This fits well with the concept of ESP modulating the host’s immune response without triggering a strong inflammatory reaction. Interestingly, ESP induced a strong upregulation of VCAM-1 in vulpine endothelial cells which was overall not evident in canine cells. This suggests that ESP may trigger immune cell recruitment in vulpine endothelial cells. Notably, only VCAM-1 showed such a strong response to ESP stimulation in foxes, while all other target genes remained close to baseline, with no significant regulation. The selective effect of ESP indicates targeted immune modulation, potentially promoting cell adhesion without initiating a comprehensive pro-inflammatory response. Foxes hence may have mechanisms to counteract the immunomodulatory strategies of ESP, thereby enabling a targeted and effective response. ESP of *S. mansoni* has been demonstrated to reduce the expression of VCAM-1 in human lung microvascular endothelial cells when these cells had been pre-activated by TNF. This mechanism might represent a strategy employed by the parasite to evade the host’s immune response by specifically reducing leukocyte recruitment to the lungs (Trottein et al., 1999). Translated to our findings, the strong upregulation of VCAM-1 induced by ESP in foxes suggests that their immune system may not be suppressed completely by these parasitic modulation mechanisms. Instead, the upregulation of VCAM-1 could enhance leukocyte recruitment, thereby supporting an effective immune response against the parasite irrespective of antibody production (Gillis-Germitsch et al., 2017). Given the well-documented role of ESP in modulating complement and inflammatory responses to facilitate the parasite’s evasion of the host’s immune defense (Lightowlers and Rickard, 1988), this is a notable finding, indicating an evolutionary adaptation in the fox host, wherein specific immune mechanisms have evolved to counteract the immunomodulatory strategies of ESP, thereby enabling a more effective response against the parasite.

The upregulation of P-selectin and ICAM-1 in fox endothelial cells after four hours suggests enhanced immune cell recruitment and a more intense inflammatory process compared to dogs. Furthermore, IL-6, a pro-inflammatory cytokine also promoting immune cell recruitment (Romano et al., 1997; Zegeye et al., 2018), was upregulated 24 hours after L1 antigen stimulation in both species, with a five-fold increase in canine endothelial cells compared to a moderate increase in fox endothelial cells. Studies on *A. cantonensis* in rats have shown that IL-6 upregulation was associated with persistent pneumonia in early infection and lung fibrosis in later stages, mediated through the IL-6/Stat3 pathway, which enhances inflammatory processes and leads to structural damage in lung tissue (Zhou et al., 2022). These findings suggest that IL-6 upregulation may also play a critical role in inflammation-associated lung damage in *A. vasorum* infections in dogs. The pronounced IL-6 upregulation in canine endothelial cells could indicate greater susceptibility to pneumonia and potentially chronic changes such as fibrosis, whereas the moderate response in fox endothelial cells suggests a lower risk of such damage.

Regarding ICAM-1, a study on *S. mansoni* in mice evidenced that ICAM-1 upregulation plays a critical role in the inflammatory response and granuloma formation by facilitating immune cell migration into affected tissues. This response helps control the infection, but prolonged ICAM-1 upregulation can lead to chronic inflammation and tissue damage, particularly fibrosis in affected organs, such as the lungs (Azevedo et al., 2012). Such changes have also been observed in the lungs of dogs and foxes infected with *A. vasorum*, where L1 cause substantial tissue damage and infiltration with inflammatory cells (Bourque et al., 2008; Gillis-Germitsch et al., 2020; Kranjc et al., 2010). The upregulation of ICAM-1 in fox endothelial cells exposed to *A. vasorum* antigens may hence represent a similar mechanism, promoting immune cell migration and accumulation to control the parasite. This enhanced immune response might allow foxes to manage the infection more effectively. In dogs, this mechanism appears less active or insufficiently effective, potentially contributing to the development of commonly observed clinical signs. Nevertheless, similar to infections with *Schistosoma* spp., prolonged ICAM-1 upregulation in foxes could contribute to lung fibrosis. A recent study illustrated that *A. vasorum*-infected foxes do exhibit notable lung pathologies at necropsy, including partial lung fibrosis, lobe consolidation, and adhesions (Gillis-Germitsch et al., 2020). This underscores the potential for long-term tissue damage caused by chronic inflammatory processes resulting from ICAM-1 upregulation.

Particularly noteworthy was the substantial upregulation of VEGF in dog cells observed after 24 hours of exposure to adult and L1 antigen, suggesting that the VEGF-mediated vascular remodeling in the lungs begins only 24 hours after stimulation. In foxes, VEGF expression yet was not changed at this time point. Furthermore, IL-33 was downregulated twofold four hours after stimulation with the adult antigen and fivefold after 24 hours. IL-33 is constitutively expressed in endothelial cells and functions as an endogenous danger signal or alarmin during tissue damage or injury, activating the immune system and triggering an inflammatory response (Cayrol and Girard, 2009; Moussion et al., 2008). Previous work demonstrated instead that VEGF reduces IL-33 expression in human endothelial cells, potentially enhancing cell responsiveness to angiogenic signals (Küchler et al., 2008). These observations align with our results, as a marked downregulation of IL-33 and simultaneous strong upregulation of VEGF were observed 24 hours post-stimulation with adult antigen. The downregulation of IL-33 may thus be a necessary step to facilitate VEGF-driven angiogenesis and the associated vascular remodeling in the lungs (Küchler et al., 2008). VEGF is the most potent pro-angiogenic stimulator, essential for angiogenesis and vasculogenesis, and increases vascular permeability, leading to enhanced exsudation of fluids and proteins into surrounding tissues (Connolly et al., 1989; Senger, 1983; Ferrara and Henzel, 1989). In *Schistosoma*-infected mice, VEGF has been shown to promote vascular remodeling in the lungs, which may contribute to the development of pulmonary hypertension (Chabon et al., 2014). The upregulation of VEGF in dogs 24 hours after stimulation therefore indicates that structural changes occur in the lungs, and, accordingly, these mechanisms could also contribute to pulmonary hypertension in dogs infected with *A. vasorum*, a clinical sign that is rare but does occur (Borgeat et al., 2015; Glaus et al., 2010; Paradies et al., 2013). Similarly, Machado et al. (2023) demonstrated that ESP and surface-associated antigens from adult *D. immitis* activate angiogenic mechanisms in an *in vitro* setting, primarily by stimulating the synthesis of proangiogenic factors. Notably, excretory-secretory antigens increased viable cell numbers, promoted cell migration, and facilitated pseudo-capillary formation. These processes may contribute to vascular endothelial remodeling in the infected host, creating a favorable environment for the parasite’s long-term survival. Conversely, VEGFS downregulation in fox endothelial cells may account for the very rare occurrence of such symptoms in this species (Philbey, 2013; Simpson, 1996; Woolsey et al., 2017). However, not observing pulmonary hypertension in foxes (and other clinical signs frequently observed in dogs) may yet also be attributable to the fact that foxes are wildlife and often harbor pathogens without showing obvious clinical signs (de Souza et al., 2019).

Our results are derived from *in vitro* experiments. In an *in vivo* situation, all three antigens are commonly present in the host system simultaneously. It hence may be plausible to assume that some of the effects observed in the present study may be less distinct on the one hand but may be more pronounced due to synergistic effects on the other hand. Moreover, it ought to be considered that the endothelial cells obtained from foxes may already have been exposed to *A. vasorum* antigens and other pathogens prevalent in foxes prior to our experiment even though donor foxes of the present work were negative for *A. vasorum* at the time the endothelial cells were harvested. All foxes were confirmed non−infected by *A. vasorum* by combined antigen and antibody ELISA screening from bloody liquid collected from the thoracic cavity (Gillis-Germitsch et al., 2017, 2020), Baermann−funnel analysis of lung tissue to isolate L1, and macroscopic examination of the lungs (Gillis-Germitsch et al., 2017, 2020). Peripheral eosinophil counts were not measured, as only thoracic cavity effusions—suitable for ELISA but not for reliable leukocyte differentials— could be obtained from hunted foxes. Moreover, eosinophilia in foxes is diagnostically poorly defined and, in canid angiostrongylosis, is not consistently present but is mostly absent (Glaus et al., 2010; Schnyder et al., 2010). Potential pre-exposure as well as the fact that our dog cell line had not been developed in-house could have influenced gene expression and the specific responses of the cells. However, we have put considerable effort into standardizing experimental conditions, and due to previous evidence that foxes have been shown not to develop any sort of adaptive immunity to *A. vasorum* (Woolsey et al., 2017), it is assumed that potential bias or memory effects can be considered of minor relevance.

While our transcriptomic analysis provides a comprehensive overview of gene regulation in canine and vulpine endothelial cells following exposure to *A. vasorum* antigens, it is important to be aware that transcript abundance does not always correlate directly with protein levels or biological activity as not all transcribed genes are translated to proteins (Bunnik et al., 2013; Lange et al., 2007). Hence, confirmation of key cytokines and adhesion molecules at the protein level (e.g., via Western blot or targeted proteomics) would provide critical functional validation. However, such experiments fall outside the primary scope of this transcriptomic-level-focused investigation. Instead, we present these expression patterns as robust transcriptional signatures that generate testable hypotheses about species‐specific immune activation and endothelial behaviour. To bridge this gap, future studies should employ immunoblotting or mass spectrometry–based approaches to verify protein expression and elucidate the mechanistic links between mRNA changes and endothelial cell function in both dogs and foxes. This integrated strategy will be essential for translating our transcriptional findings into a comprehensive understanding of host‐parasite interactions.

## 5 Conclusion

Our findings indicate that host response varies by antigen type, timing, and host species. *Angiostrongylus vasorum* ESP elicited a mild immune response, highlighting its immunomodulatory role, while adult and L1 antigens triggered stronger inflammation. Dog cells showed a delayed but less regulated response, potentially contributing to tissue damage and, accordingly, supporting the onset of clinical signs. In contrast, fox cells responded quickly and diversely, stabilizing endothelial activation after 24 hours, indicating that foxes possess mechanisms enabling them to control the parasite more effectively by consequently avoiding severe consequences for the host.

These results contribute to the evidence that parasite-host co-evolution has shaped immune responses in the wildlife reservoir, influencing disease pathogenesis. Red foxes may have developed mechanisms to tolerate parasite presence mostly without severe clinical consequences. In contrast, in dogs the co-evolutionary process between parasite and host appears to be less advanced, as canine angiostrongylosis remains a severe and potentially fatal disease.

## Data Availability

The datasets presented in this study can be found in the article/**Supplementary Material**.
