# Supplementary material for: Host-specific vascular endothelial cell responses to Angiostrongylus vasorum: a comparative in vitro study in red foxes (Vulpes vulpes) and domestic dogs
Source: Front Cell Infect Microbiol. 2025 Jun 3;15:1584663. doi: 10.3389/fcimb.2025.1584663 (PMC12170705; doi:10.3389/fcimb.2025.1584663)
Supplement: Supplementary file 1 [file DataSheet1.docx]

Supplementary Material

# Supplementary Figures

**Supplementary Figure 1.** Immunohistochemical visualization of canine platelet endothelial cell adhesion molecule-1-positive primary endothelial cells.


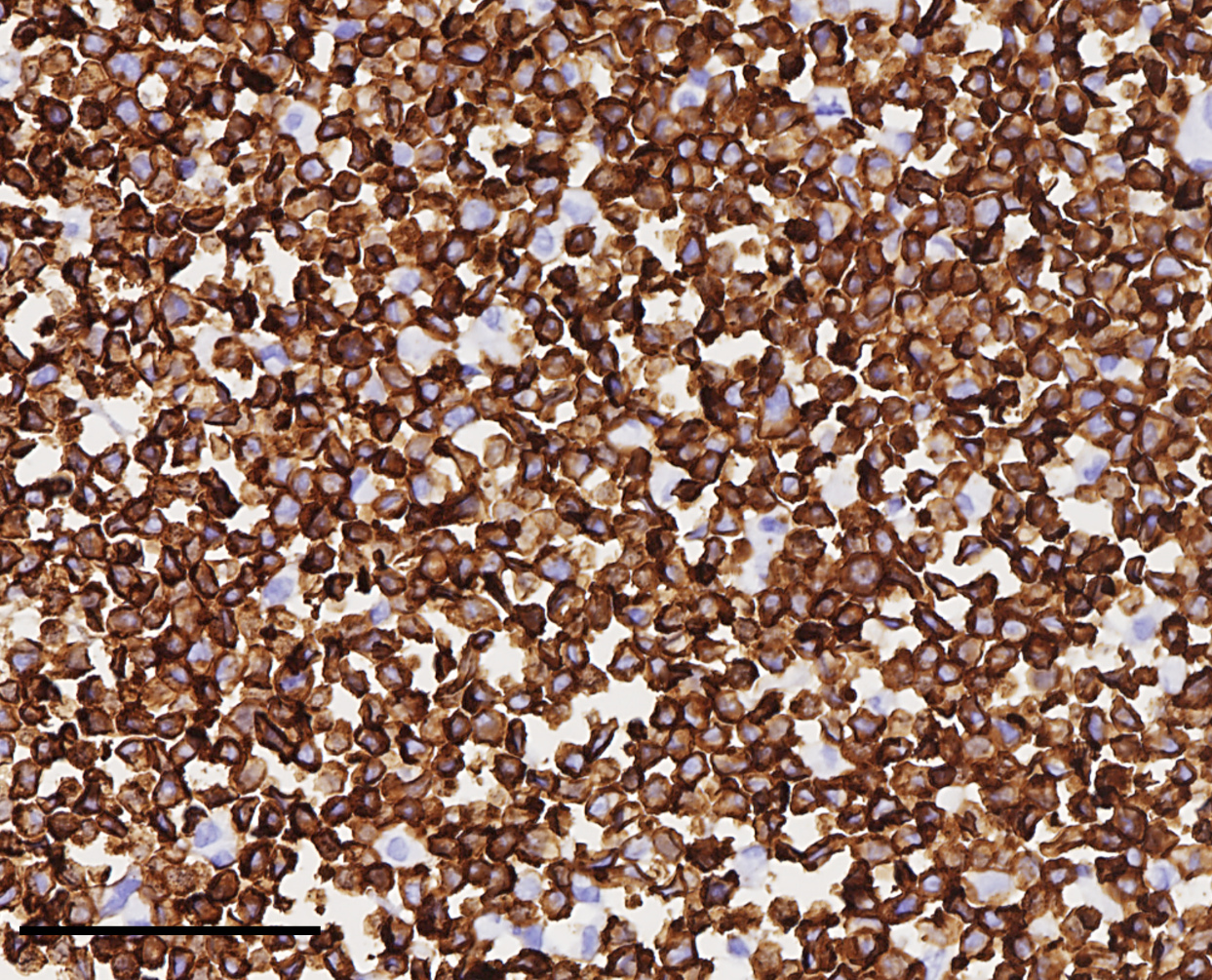


Scale bar: 100 µm.
